# Investigation of CVD graphene as-grown on Cu foil using simultaneous scanning tunneling/atomic force microscopy

**DOI:** 10.3762/bjnano.9.274

**Published:** 2018-11-28

**Authors:** Majid Fazeli Jadidi, Umut Kamber, Oğuzhan Gürlü, H Özgür Özer

**Affiliations:** 1 Department of Physics Engineering, İstanbul Technical University, 34469, İstanbul, Turkey

**Keywords:** atomic force microscopy, CVD graphene, scanning tunneling microscopy, simultaneous operation, small amplitude

## Abstract

Scanning tunneling microscopy (STM) and atomic force microscopy (AFM) images of graphene reveal either a triangular or honeycomb pattern at the atomic scale depending on the imaging parameters. The triangular patterns at the atomic scale are particularly difficult to interpret, as the maxima in the images could be every other carbon atom in the six-fold hexagonal array or even a hollow site. Carbon sites exhibit an inequivalent electronic structure in HOPG or multilayer graphene due to the presence of a carbon atom or a hollow site underneath. In this work, we report small-amplitude, simultaneous STM/AFM imaging using a metallic (tungsten) tip, of the graphene surface as-grown by chemical vapor deposition (CVD) on Cu foils. Truly simultaneous operation is possible only with the use of small oscillation amplitudes. Under a typical STM imaging regime the force interaction is found to be repulsive. Force–distance spectroscopy revealed a maximum attractive force of about 7 nN between the tip and carbon/hollow sites. We obtained different contrast between force and STM topography images for atomic features. A honeycomb pattern showing all six carbon atoms is revealed in AFM images. In one contrast type, simultaneously acquired STM topography revealed hollow sites to be brighter. In another, a triangular array with maxima located in between the two carbon atoms was acquired in STM topography.

## Introduction

Graphene has been widely studied because of its potential use in future nanoelectronics, as it provides unprecedented mobility of charge carriers at room temperature [1]. Moreover, very high conductivity at room temperature and a half-integer quantum Hall effect suggest the presence of relativistic charge carriers with vanishing mass [2]. Graphene has been investigated by using scanning tunneling microscopy (STM) and atomic force microscopy (AFM) by various groups [3]. The interaction of graphene with its substrate affects the STM measurements and that casts doubts on its electronic structure. Having the possibility to make simultaneous STM and AFM measurements, on the same area would be useful in understanding the mechanisms of such interactions.

Nowadays, a variety of methods are used to prepare graphene. Mechanical exfoliation of graphite facilitates obtaining micrometer-scale graphene layers on amorphous substrates such as silicon oxide [1]. Graphene monolayers have been grown on metallic surfaces by thermal decomposition of hydrocarbons. Graphene layers on metallic substrates can be transferred on to other substrates by wet-chemical methods [4].

Theoretically, current in STM and attractive force in AFM have a similar origin [5]. However, STM behavior is more complicated as it could be interpreted as a map of the local charge density of states of the surface at the Fermi level [6]. Therefore, depending on tip type and its electronic charge state, different relative contrasts of the atoms on the surface, including even reversal of contrast, are obtained in STM images. In AFM images as well, tip structure and force/force gradient regime may result in different relative contrasts at the atomic scale.

During the past decade, STM and AFM studies of graphene have been shown to yield honeycomb or triangular patterns due to different experimental parameters or sample preparation [7–10]. Also, large hexagonal periodic arrays, known as moiré patterns, with periodicities of several nanometers were reported [10–13]. These periodic moiré patterns were generally attributed to rotational misorientation of layers, lattice mismatch or subsurface defects. Despite numerous investigations and the simplicity of the honeycomb structure of graphene, interpreting the maxima points in triangular patterns obtained in STM or AFM images as atoms or hollow sites still remains a challenging problem [14–17]. The honeycomb pattern images are interpreted as images of all six carbon atoms, whereas the triangular pattern of bright spots is interpreted as an image of three of six carbon atoms or of no carbon atoms at all, but of hollow sites.

In most of the STM investigations on graphene, atomic triangular patterns were reported for a broad range of bias voltages [18–21]. In addition, some groups have reported honeycomb patterns obtained at low bias voltages [22–24]. Bernal stacking is supposed to be the most reliable hypothesis for the observation of triangular patterns. In this configuration, due to the shift between two successive layers, three carbon atoms directly align with the carbon atoms of the graphene layer below, which are denoted as *a* atoms. The other three carbon atoms, which are denoted as *b* atoms, are placed right above the hollow sites of the underlying graphene layer. This results in the two types of surface atoms to be electronically inequivalent. Since, *b* atoms have a greater contribution to the density of states close to Fermi energy compared to *a* atoms, they are imaged as bright spots at low bias voltages based on the STM investigations. These *b* atoms form a new larger triangular structure in STM images (two lattice points in this hexagonal structure are spaced √3-times the typical neighboring C–C bond length, which means a unit cell length of about 0.246 nm). Although most of the STM studies on graphene at low bias voltages show triangular patterns where *b* atoms were indicated as bright spots, some other experiments at closer tip–sample separations show triangular patterns where bright spots were identified as hollow sites. This is interpreted as a consequence of current saturation in the near-contact regime [25–26].

There is a variety of substrate materials which graphene is grown on. Graphene–metal bonds could be divided into two main groups, i.e., “strongly bonded” and “weakly bonded”. Graphene–Ni, graphene–Co and graphene–Rh are classified as strongly bonded and others such as graphene–Cu, graphene–Au and graphene–Ir are classified as weakly bonded. Moreover, graphene–metal interaction can result in a lattice-matched (graphene–3d metals) or lattice-mismatched configuration (graphene–4d/5d metals) [10].

Material and termination of the apex of the tip play an important role in STM and AFM results as well. Due to the strong interaction between metal tip and carbon atoms, atomic resolution can be obtained in both attractive and repulsive regimes, but with inverted contrast [9]. Some FM-AFM studies using Si tips on graphite(0001) show a triangular pattern of bright spots instead of a honeycomb pattern [27]. Researchers have been trying to observe the three hidden carbon atoms. Hembacher et al. presented results showing the three missing atoms at low temperature (4.89 K) with AFM using an STM/AFM setup they developed [7]. However, not all of the six carbon atoms could be observed with STM in that study.

In this work, using a simultaneous STM/AFM working with sub-angstrom oscillation amplitudes we intended to gain insight on relative contrast mechanisms in STM and AFM, on a graphene surface. In the past, we used this small-amplitude STM/AFM technique in order to investigate surfaces such as Si(100) [28], Cu(100) [29], Si(111) [30], and more recently Si(111) again, with an improved force resolution [31].

## Experimental

Our setup is a commercial STM/AFM operated in ultrahigh vacuum (UHV) that was modified and improved by the implementation of a Fabry–Pérot fiber interferometer in order to achieve high sensitivity in detecting lever deflection and measuring the oscillation amplitude, which in turn allows us to conduct simultaneous STM/AFM measurements. This system with a sensitivity of 2·10^−4^ Å/√Hz is capable of measuring tunnel current, force, force gradient, tunnel barrier height and energy loss [32]. The UHV chamber is equipped with an argon sputtering gun and a resistive heater that could be used for sample–tip preparation. The main chamber is pumped with a combination of an ion getter pump, titanium sublimation pump and a turbomolecular pump backed with a double-stage rotary pump. A base pressure of about 10^−10^ mbar is achievable by baking out the chamber.

The oscillation amplitude of the cantilever plays a major role for the actual simultaneous measurement of forces and tunneling current. Large lever oscillation amplitudes offer only a limited range in which tip is in the tunneling cycle. Using very small (sub-angstrom) lever oscillation amplitudes, the tip is kept in the tunneling range during the entire cycle. Consequently, while the constant tunnel current is used as feedback loop, the use of very small oscillation amplitudes ensures simultaneous STM/AFM operation as close as possible to the actual STM mode [30,33]. The combination of these two techniques attracts great interest for conducting or semiconducting samples, because the acquired spectroscopies associated with forces and tunneling current between the foremost atoms of probe and sample provide complementary results. This would be helpful to detect distinct unique structures or chemical adsorbates on surfaces or in nanostructures.

With the use of sub-angstrom oscillation amplitudes, far from resonance, the tip–sample force corrugation in the images obtained at very close separations, can be calculated using *F* = *k*_lever_·(*A*_0_ – *A*), where *k*_lever_ is the lever stiffness, *A*_0_ the free oscillation amplitude, i.e., far from the surface, and *A* is the measured amplitude during tip–surface interactions [34].

Graphene layers were grown on Cu foils using chemical vapor deposition (CVD) [35]. A custom-built atmospheric CVD system was used. Cu foils were heated under H_2_ + Ar atmosphere up to 950 °C. Upon reaching the process temperature, the Ar flow was stopped and the H_2_ flow was reduced while CH**_4_** was let in to the quartz tube as the carbon source. As-grown samples, as well as graphene crsytals transferred on to dielectric substrates were investigated by optical microscopy and scanning electron microscopy (SEM). Raman spectra taken on several samples showed the presence of dominantly single-layer graphene [36].

## Results and Discussion

**Figure 1:**
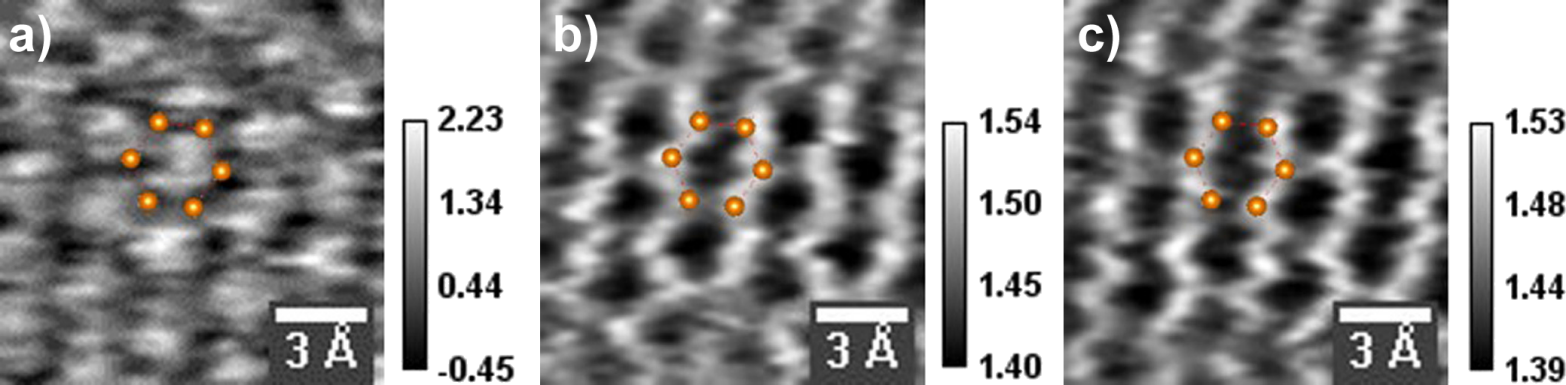
Simultaneous STM/AFM images of a graphene surface on a Cu substrate. (a) STM topography in constant-current mode (forward scan); (b) force in constant-current mode (forward scan); (c) force in constant-height mode (backward scan). Scale bar values are in units of Å in STM and nN in force images. Obtained with a W tip and cantilever of stiffness *k* = 53 N/m and a resonance frequency *f*_0_ = 31.5 kHz; drive frequency *f* = 15 kHz. The image size is 11 × 11 Å^2^, *V*_sample_ = −0.5 V, *I*_t_ = 1.2 nA, free amplitude *A*_0_ = 0.3 Å_rms_. A hexagonal unit cell is superimposed.

We used a custom-made tungsten tip–cantilever probe [32] the stiffness of which was estimated from thermal oscillations to be about 53 N/m. The simultaneously acquired STM topography and force images of a graphene surface are shown in Figure 1. In this experiment, the forward scan was performed in constant current mode and the backward scan in constant height mode, for a proper extraction of the tip–sample force, which might have been potentially affected by the varying tip trajectory. STM topography in Figure 1a was obtained in the forward scan in constant-current mode and the other two (Figure 1b,c) are the forward (constant current) and backward (constant height) scans of the force, respectively. A honeycomb pattern is clearly seen in force images. All six carbon atoms are visible. The STM image shows a triangular pattern, the inversion of which is a honeycomb structure. This means that hollow sites are imaged with higher contrast than the carbon atoms. This was predicted to be possible due to saturation of tunnel current over carbon atoms at relatively small tip–surface distances [25]. The same area scanned with the same parameters except for the opposite sample bias of 500 mV resulted in an inversion of STM contrast, showing the honeycomb structure where C atoms are higher in contrast than the hollow sites.

In the force image, brighter shading corresponds to more positive values. This means that C atoms reveal a more repulsive force compared to the hollow sites. This makes sense if the imaging regime is beyond the force minimum (i.e., the tip is closer to the surface than the minimum of the force). This will be supported by the force–distance (*F*–*d*) measurement presented below. Ondracek et al. [25] reported a comprehensive theoretical study of the contrast mechanisms in AFM of carbon surfaces with different tip structures (different terminations of Si and metal tips). The force curves they calculated using a tungsten tip show the hollow sites to be more attractive beyond the force minimum of both sites. Our results are consistent with these calculations. The use of pure metallic tips is not common in non-contact AFM measurements, since the widely used micro-fabricated levers are made of Si and at best these are coated with metals such as Pt/Ir in order to obtain a metallic apex. However, the uncertainty whether there is sufficient coating at the very apex of the tip or not and whether that coating remains during the experiments casts doubt on the metallic nature of the tip. Only in a limited number of experiments where a tuning fork is used as the sensing element, W tips are attached to the end of the sensor. The use of metallic tips is particularly crucial in simultaneous measurement of tunnel current and force.

Force values are calculated from oscillation amplitude data using the equation above for carbon and hollow sites. This was done for both constant-current (CC) and constant-height (CH) scans. The force corrugation contrast between carbon and hollow sites is found to be about 0.1 nN for both CC and CH modes. Thus, as far as the force is concerned the tip trajectory in CC mode does not have a considerable influence on the measurement of the oscillation amplitude. A similar behavior was observed in a previous work on Si(111) using the same small amplitude STM/AFM technique [37].

**Figure 2:**
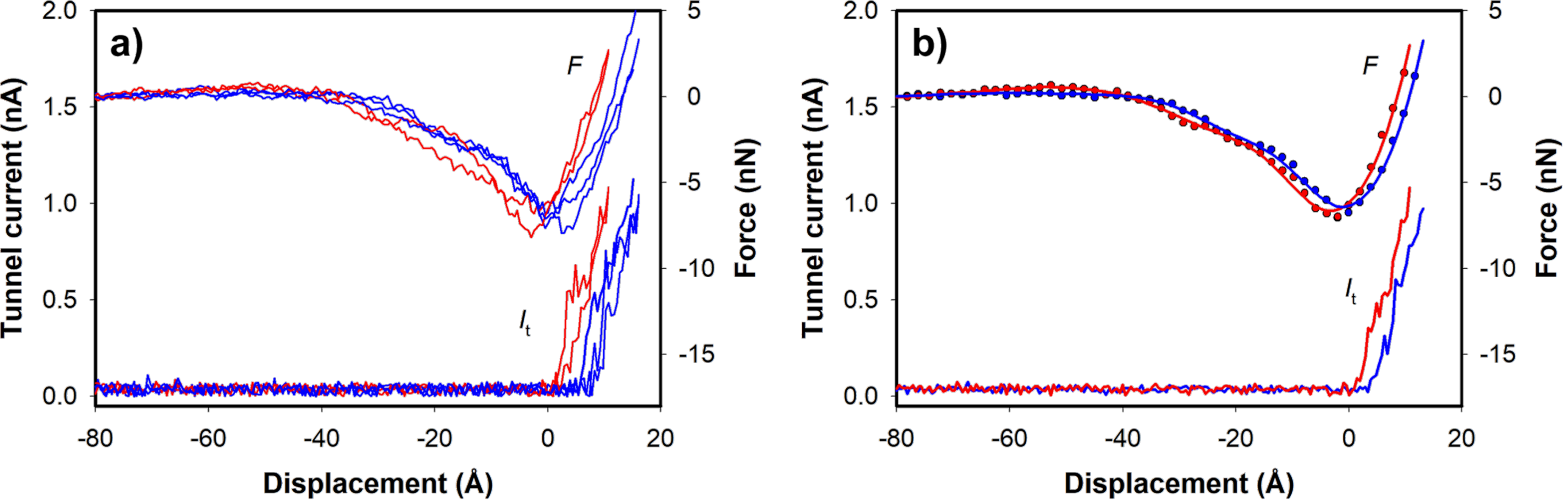
*F*–*d* spectroscopy; force (*F*) and tunnel current (*I*_t_) were measured as functions of the relative tip–sample displacement. a) Several curves on carbon (red) and hollow sites (blue) showing two distinct features. b) Average of curves on carbon (red) and hollow sites (blue). Solid lines in force curves are smoothened raw data (dotted). The curves at the hollow site are displaced by 2 Å to account for the shift in position of carbon and hollow sites. The curves were obtained using the W cantilever used in the imaging experiments, with *k* = 53 N/m; *V*_sample_ = −0.35 V.

As mentioned before, the tip–sample distance (interaction regime) plays an important role in the relative contrast of different sites on the graphene surface. In order to shed light on the contrast differences in imaging we have also conducted force–distance (*F*–*d*) spectroscopy measurements. At a certain point on the sample, by changing the distance between the surface and the tip, we simultaneously measure the force as well as tunnel current. It is difficult to do site-specific spectroscopy at room temperature due to thermal drift. Our goal was to carry out spectroscopy on a carbon and a hollow site. We imaged the surface for a long while until the thermal drift was minimized. Then by moving the tip to targeted lateral positions (carbon and hollow sites) we obtained several *F*–*d* curves. The curves revealed two distinct features particularly regarding the onset positions of tunnel current and force interaction, as shown in Figure 2a. With this statistical approach we were confident that the curves were taken at carbon and hollow sites. Since we start the *F*–*d* measurements at a particular tunnel-current set point, the curves obtained on the hollow site are displaced by 2 Å, obtained from the STM corrugation in the image before, to account for the shift between the hollow and carbon site measurements (Figure 2b). Based on various force–distance spectroscopy measurements acquired with different parameters and different tips, almost in all of the cases our images were obtained while operating in the regime beyond maximum attractive force. The force curves show, as expected, the initial attractive forces and the repulsive forces after the minimum is reached. The maximum attractive force is about 7 nN for both sites. Ondracek et al. calculated the maximum attractive force between a tungsten tip and carbon and hollow sites on graphene to be about 2 nN [25]. However, in their calculations they took into account the short-range interaction and the longer-range van der Waals (vdW) interaction for a small tip structure only, but not the electrostatic force. The discrepancy in the value of the maximum attractive force and the interaction range of the total measured force, which are much larger than the values derived from the theoretical calculations, is explicable with the additional effect of electrostatic forces in the experiments. The sample bias voltage values used in the experiments (up to 500 mV) would result in considerable electrostatic force between the tip and the sample. Also, tips with relatively large cone angles would result in van der Waals interactions with a larger range. When we compare the range of interactions in the tunnel current and force measurements, we see a different situation to that reported on other surfaces in previous studies. Based on previous works on semiconductors such as Si(111) and Si(100), the onset of tunnel current is usually before the maximum attractive force interaction. So, the typical tunnel current value used in the STM enables one to study in both the attractive and repulsive force region. The situation in graphene surface measurements is quite different. The force interaction starts well before the onset of the tunneling current. In our simultaneous STM/AFM experiments, we used the tunnel current to control the tip–surface distance. Evidently, tunnel currents as small as 0.1–0.2 nA keep the interaction beyond the minimum of the force curve.

**Figure 3:**
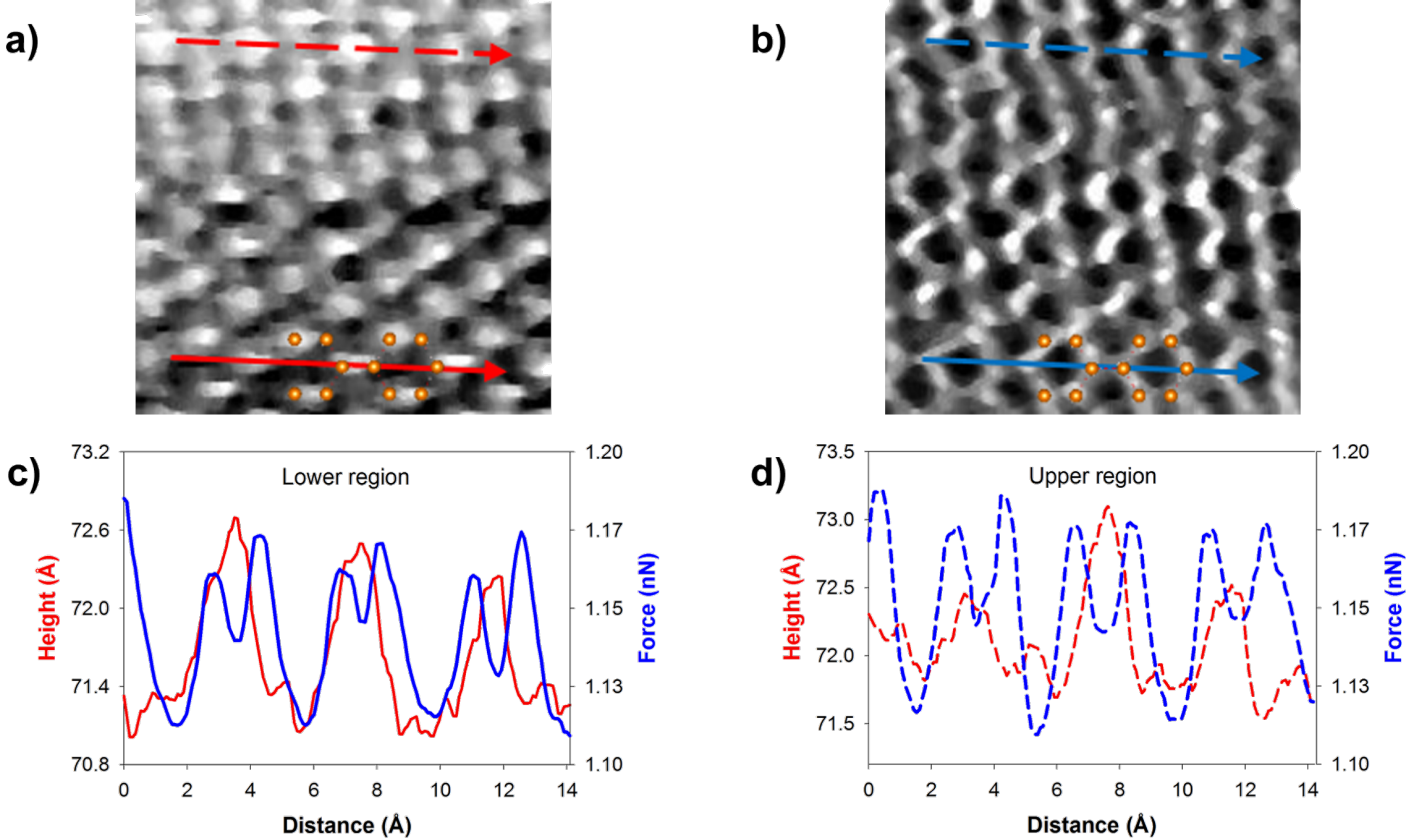
Simultaneous STM/AFM images of a graphene surface. (a) STM topography in constant-current mode (forward scan); (b) force in constant-height mode (backward scan). A pair of hexagonal unit cells is superimposed on the images. The profiles along the indicated lines are given in panels (c) and (d). Images obtained with a W cantilever of stiffness *k* = 53 N/m and a resonance frequency *f*_0_=31.5 kHz; drive frequency *f* = 15 kHz. The image size is 18 × 18 Å^2^; *V*_sample_ = 0.5 V, *I*_t_ = 0.6 nA, free amplitude *A*_0_ = 0.3 Å_rms_.

Another set of simultaneous scans obtained using the same W cantilever at a positive sample bias of +0.5 V is shown in Figure 3. The tunnel-current set point of 0.6 nA is smaller than the value used for the image in Figure 1. Hence, we expect a relatively weaker force interaction between the tip and sample. In this figure, the STM topography obtained in the forward scan in constant-current mode and the force image acquired in backward scan in constant-height mode are shown. The force measurement shows the honeycomb pattern in the entire image with some local distortions throughout the image. On the other hand, the STM topography exhibits a triangular pattern with a slight difference in two different regions. Also, there is a small gradual increase in height starting midway through the STM image, which suggests a change in overall local density of states (LDOS) or topography. Line profiles of the force and STM topography in the two regions are given in the figure.

In the lower part of the scan, the line profile of the force clearly distinguishes the two C atoms and the hollow sites, one of the C atoms revealing a considerably larger force than the other. Comparing the STM line scan with the force, we see that the bright spots in the triangular pattern of STM topography corresponds to the middle of the two C atoms. The observation of STM topography maxima between *a*-type and *b*-type carbon atoms in HOPG is predicted to be possible in the work by Teobaldi and co-workers [38]. Upon obtaining remarkable contrast variations with the bias voltage in low-temperature STM experiments, the authors calculated and discussed the role of bias voltage and tip termination on the atomic contrast in constant tunnel current images of HOPG. They chose different terminations of W tips and different relative orientations of the tip and graphite surface. Over a wide range of bias voltages, they have shown the transitions between different contrast types. Their calculations suggest that, sharp W tips can reveal maxima between *a*-type and *b*-type carbon atoms at bias voltages between 0.3 and 0.7 V. In the upper part of the scans, we see a qualitative behavior similar to the lower part in both STM and force. However, the line profiles show a different quantitative behavior in force scan. Now the two C atoms reveal the same force value as can be clearly seen in the line profile. It is well known in scanning probe microscopy that such contrast changes may occur due to a change in the atomic structure of the tip apex. The fact that there is no sign of an abrupt change in the scans in both channels and the gradual increase in height in STM scan, which amounts up to 1 Å, may have suggested a real height change due to buckling/twisting of the topmost layer or change in LDOS between the two regions. The change in force contrast accompanied by a slight change in STM topography could suggest a change in alignment of the topmost layer with the underlying graphene layer or the substrate. However, we cannot rule out the possibility of a tip change occurring smoothly during several scan lines, which would result in the changes observed in force and STM images, both qualitatively and quantitatively.

## Conclusion

We investigated graphene grown on Cu foils using simultaneous STM/AFM with a metallic cantilever oscillated with sub-angstrom amplitudes. Different relative contrasts between STM and force are obtained. Both honeycomb and triangular patterns are observed in STM images. The maxima in the triangular patterns in STM images of HOPG and graphene presented in the literature have been assigned to either *a*-type or *b*-type carbon atoms or hollow sites. The triangular patterns in STM topography in this work resulted in maxima in between the two C atoms, which is supported by theoretical calculations. Such an observation would not be possible without simultaneous acquisition of tunnel current and force interaction. Under the typical STM imaging conditions of graphene, the tip–sample interaction force is in repulsive regime. This behavior, which has been observed ever since the very early STM results of the HOPG surface, is shown to be the case in graphene surface as well. Hence atomic relaxations might be quite influential in STM imaging of graphene. The contrast mechanisms could be understood using simultaneous STM/AFM. Truly simultaneous operation is possible only with the use of sub-angstrom oscillation amplitudes.

## References

[1] Novoselov K S, Geim A K, Morozov S V, Jiang D, Zhang Y, Dubonos S V, Grigorieva I V, Firsov A A (2004). Science.

[2] Varykhalov A, Sánchez-Barriga J, Shikin A M, Biswas C, Vescovo E, Rybkin A, Marchenko D, Rader O (2008). Phys Rev Lett.

[3] Dedkov Y, Voloshina E, Fonin M (2015). Phys Status Solidi B.

[4] Geim A K, Novoselov K S (2007). Nat Mater.

[5] Chen C J (1991). J Phys: Condens Matter.

[6] Tersoff J, Hamann D R (1983). Phys Rev Lett.

[7] Hembacher S, Giessibl F J, Mannhart J, Quate C F (2005). Phys Rev Lett.

[8] Sun Z, Hämäläinen S K, Sainio J, Lahtinen J, Vanmaekelbergh D, Liljeroth P (2011). Phys Rev B.

[9] Boneschanscher M P, van der Lit J, Sun Z, Swart I, Liljeroth P, Vanmaekelbergh D (2012). ACS Nano.

[10] Dedkov Y, Voloshina E (2014). Phys Chem Chem Phys.

[11] Yıldız D, Gürlü O (2016). Mater Today Commun.

[12] Ouseph P J (2000). Appl Surf Sci.

[13] Sun H-L, Shen Q-T, Jia J-F, Zhang Q-Z, Xue Q-K (2003). Surf Sci.

[14] Hölscher H, Allers W, Schwarz U D, Schwarz A, Wiesendanger R (2000). Phys Rev B.

[15] Hembacher S, Giessibl F J, Mannhart J, Quate C F (2003). Proc Natl Acad Sci U S A.

[16] Ashino M, Schwarz A, Behnke T, Wiesendanger R (2004). Phys Rev Lett.

[17] Albers B J, Schwendemann T C, Baykara M Z, Pilet N, Liebmann M, Altman E I, Schwarz U D (2009). Nat Nanotechnol.

[18] Binnig G, Fuchs H, Gerber C, Rohrer H, Stoll E, Tosatti E (1986). Europhys Lett.

[19] Park S-I, Quate C F (1986). Appl Phys Lett.

[20] Cisternas E, Stavale F, Flores M, Achete C A, Vargas P (2009). Phys Rev B.

[21] Mizes H A, Park S-i, Harrison W A (1987). Phys Rev B.

[22] Ouseph P J, Poothackanal T, Mathew G (1995). Phys Lett A.

[23] Paredes J I, Martínez-Alonso A, Tascón J M D (2001). Carbon.

[24] Wang Y, Ye Y, Wu K (2006). Surf Sci.

[25] Ondráček M, Pou P, Rozsíval V, González C, Jelínek P, Pérez R (2011). Phys Rev Lett.

[26] Blanco J M, González C, Jelínek P, Ortega J, Flores F, Pérez R (2004). Phys Rev B.

[27] Allers W, Schwarz A, Schwarz U D, Wiesendanger R (1999). Appl Surf Sci.

[28] Özer H Ö, Atabak M, Ellialtıoğlu R M, Oral A (2002). Appl Surf Sci.

[29] Özer H Ö, Norris A, Oral A, Hoffmann P M, Pethica J B (2004). Nanotechnology.

[30] Oral A, Grimble R A, Özer H Ö, Hoffmann P M, Pethica J B (2001). Appl Phys Lett.

[31] Özgür Özer H (2019). Ultramicroscopy.

[32] Oral A, Grimble R A, Özer H Ö, Pethica J B (2003). Rev Sci Instrum.

[33] Herz M, Schiller C, Giessibl F J, Mannhart J (2005). Appl Phys Lett.

[34] Matei G, Jeffery S, Patil S, Khan S H, Pantea M, Pethica J B, Hoffmann P M (2008). Rev Sci Instrum.

[35] Li X, Cai W, An J, Kim S, Nah J, Yang D, Piner R, Velamakanni A, Jung I, Tutuc E (2009). Science.

[R36] 36Kamber, U.; Kıncal, C.; Yagci, M. B.; Birer, O.; Gürlü, O. *Langmuir submitted for publication ***2018***.*

[37] Özer H Ö, O’Brien S J, Pethica J B (2007). Appl Phys Lett.

[38] Teobaldi G, Inami E, Kanasaki J, Tanimura K, Shluger A L (2012). Phys Rev B.

